# Supplementary material for: Effect of Drying Methods on the Morphological and Functional Properties of Cellulose Ester Films
Source: Polymers (Basel). 2025 Nov 14;17(22):3026. doi: 10.3390/polym17223026 (PMC12656642; doi:10.3390/polym17223026)
Supplement: Supplementary file 1 [file polymers-17-03026-s001.zip › polymers-3919472-supplementary.pdf]

Supplementary Information

Effect of Drying Methods on the Morphological and Functional Properties of Cellulose Ester Films

Tanuj Kattamanchi [1*], Heikko Kallakas [1], Elvira Tarasova [2], Percy Festus Alao [1], Andres Krumme [2], and Jaan Kers [1]

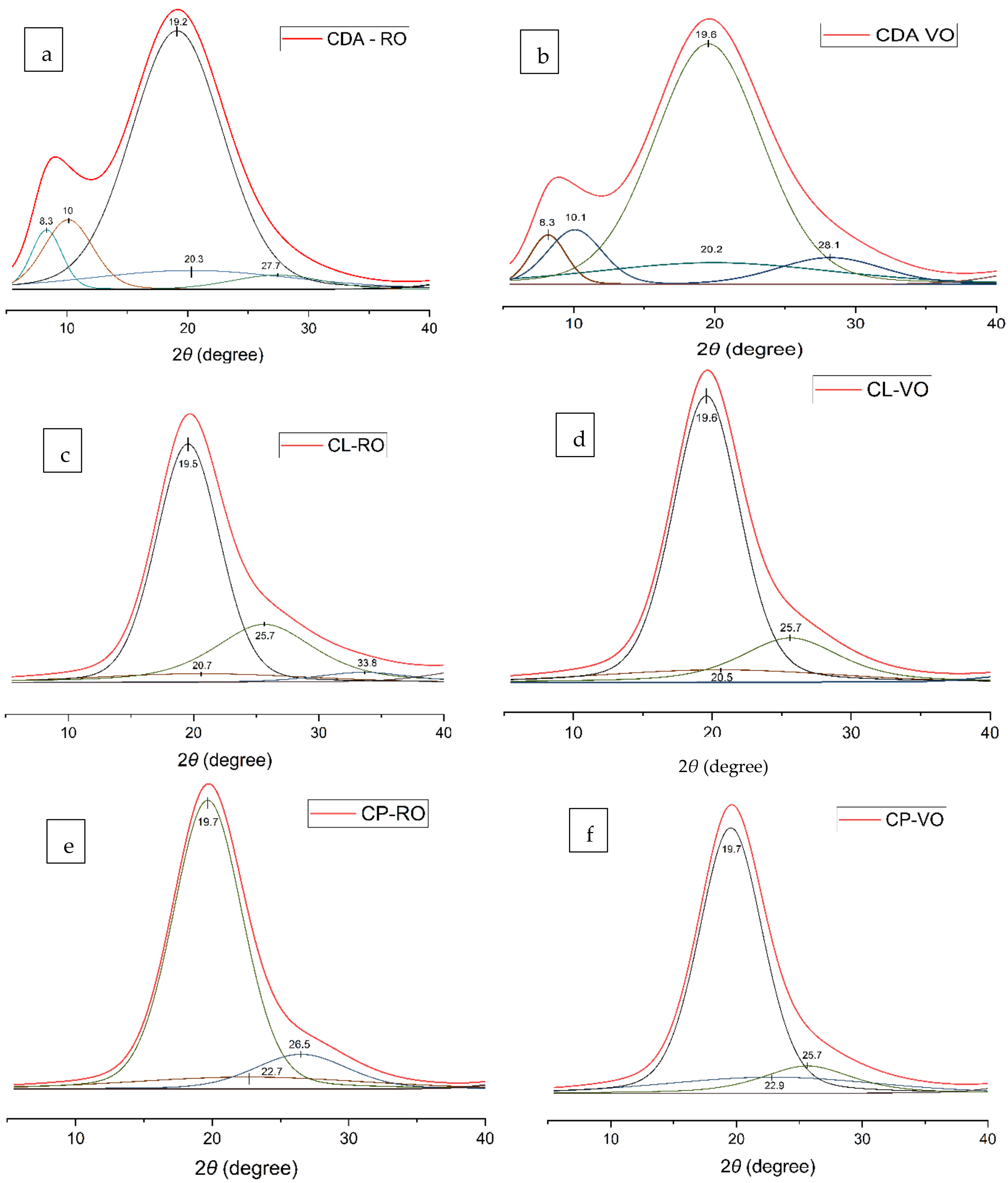

Figure S1: XRD patterns of cellulose esters images of (a-b) CDA, (c-d) CL, and (e-f) CP

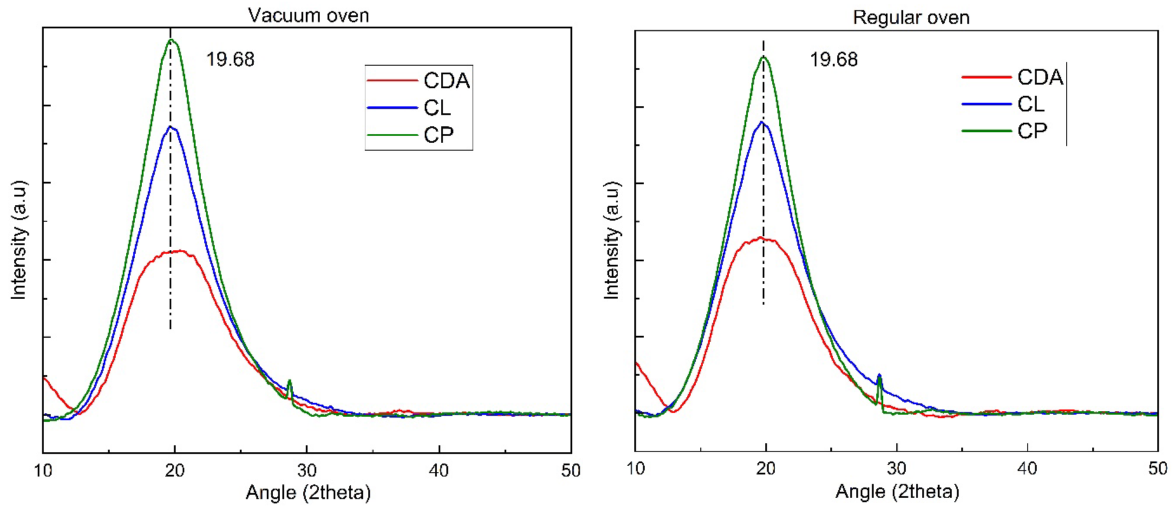

Figure S2. XRD pattern of cellulose esters dried in VO and RO

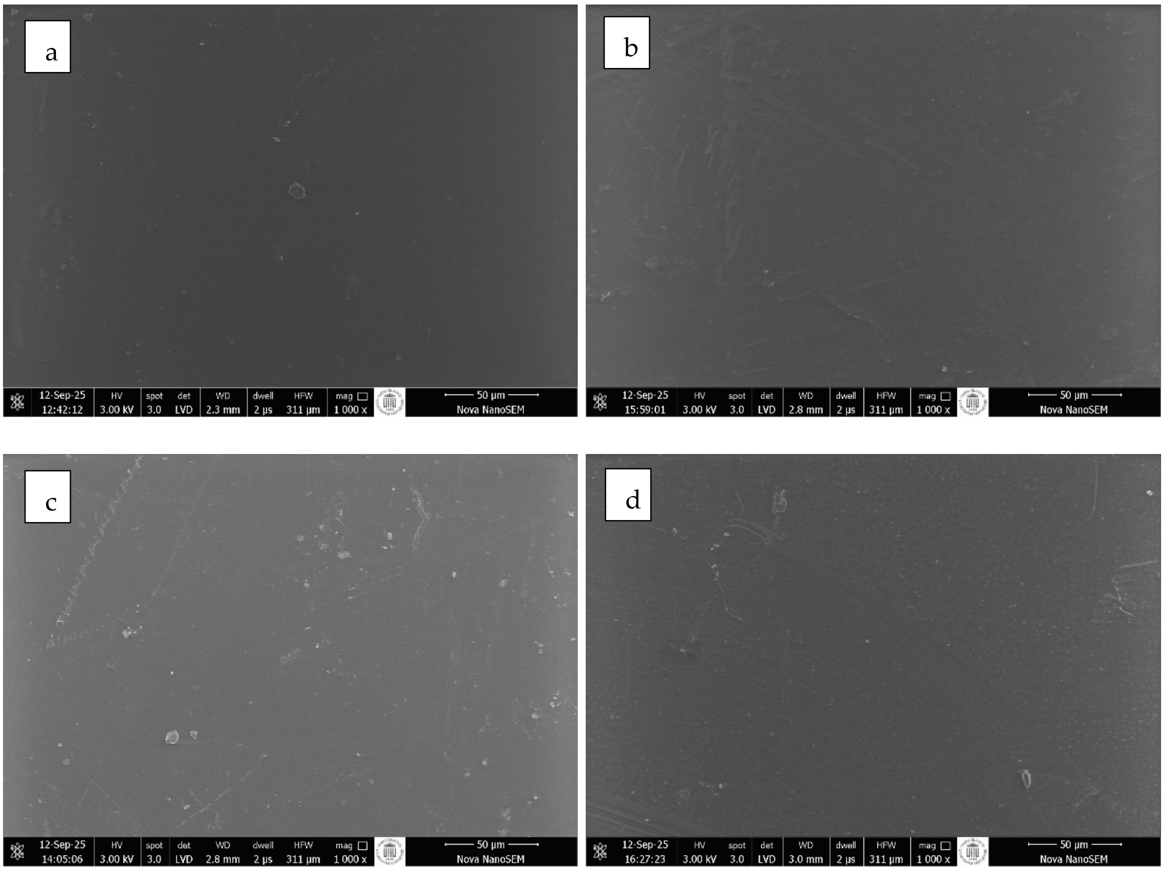

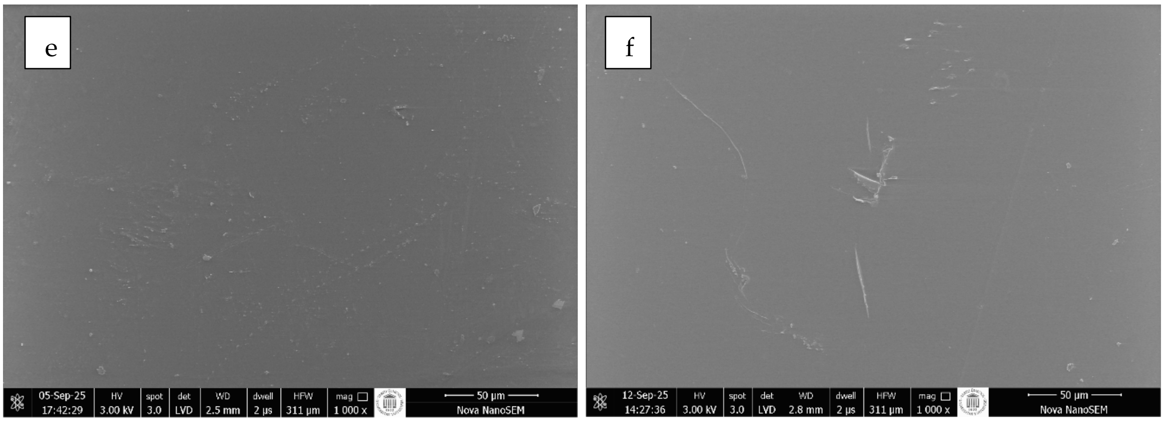

Figure S3. SEM surface images of cellulose esters are magnification of 1000x a) CDA-RO, b) CDA-VO, c) CL-RO, d) CL-VO, e) CP-RO, and f) CP-VO.
